# A Major Facilitator Superfamily Transporter Is Critical for the Metabolism and Biogenesis of the Apicoplast

**DOI:** 10.3390/pathogens14080763

**Published:** 2025-08-01

**Authors:** Yumeng Liang, Wei Qi, Jiawen Fu, Honglin Jia

**Affiliations:** 1 State Key Laboratory for Animal Disease Control and Prevention, Harbin Veterinary Research Institute, Chinese Academy of Agricultural Sciences, Harbin 150069, China; liangyumeng@caas.cn (Y.L.); q104892@163.com (W.Q.); fu.jiawen@foxmail.com (J.F.); 2 Heilongjiang Research Center for Veterinary Biopharmaceutical Technology, Harbin Veterinary Research Institute, Chinese Academy of Agricultural Sciences, Harbin 150069, China

**Keywords:** *Toxoplasma gondii*, apicoplast, MFS transporter, metabolic activity, biogenesis

## Abstract

The apicoplast is a highly specialized organelle in the biosynthesis of essential metabolites in most of the apicomplexan protozoa. This organelle is surrounded by four layers of membranes. However, the molecular mechanisms mediating transmembrane transport are not yet fully understood. In this study, we conducted a phenotypic analysis to investigate the role of a major facilitator superfamily transporter (TgApMFS1) in the survival of the parasite. The results indicated that TgApMFS1 is critical for the survival of *Toxoplasma gondii* in cell culture conditions. Further analysis indicated that these transporters are crucial for the biogenesis of organelles and the metabolic processes of parasite.

## 1. Introduction

Toxoplasmosis is an opportunistic protozoan disease. *Toxoplasma* infections are widespread in humans and other animals across all continents [1]. While the infection usually has no obvious clinical symptoms in healthy people, it may cause pathological changes such as encephalitis, and non-specific clinical signs, such as fever, rash, and atrophy. Infection of pregnant women with *Toxoplasma gondii* may lead to miscarriage during pregnancy [2] or cause congenital toxoplasmosis in the fetus. This disease could cause severe problems for individuals infected with HIV or those who have undergone organ transplantation.

The apicoplast is an organelle that originated from a secondary endosymbiotic event of apicomplexan parasites. This organelle is located near the nucleus of the parasite and has an independent structure with a diameter from 0.15 to 1.5 μm. Like non-green plastids, the apicoplast houses several essential metabolic pathways. These include the synthesis pathways for heme, type II fatty acids (FA), and lysophosphatidic acid (LPA), as well as the methylerythritol phosphate (MEP) pathway, which is crucial for producing isopentenyl pyrophosphate (IPP) [3,4]. The synthesis of fatty acids and lysophosphatidic acid in the apicoplast provides substrates needed for lipid synthesis in the endoplasmic reticulum (ER). The isoprenoids generated in the apicoplast serve as precursors for ubiquinone synthesis. Additionally, central carbon metabolism in the apicoplast also plays a role in supplying reducing equivalents and nucleotides.

The apicoplast is covered with four layers of membranes. However, the mechanisms of metabolite transport across these membranes remain largely unknown even in entire apicomlexan parasites. For a long time, only phosphate transporters of *T. gondii* (TgAPT1) and *Plasmodium falciparum* (PfiTPT and PfoTPT) were reported in the literature [5,6]. These transporters are capable of supplying phosphorylated carbons [5,7,8,9,10]. The function of a two-pore channel was only recently identified in *T. gondii* [6]. More recently, the function two novel monocarboxylate transporters [11,12] and a major facilitator superfamily transporter [13] in the metabolism of the apicoplast is investigated in *T. gondii*.

In this study, a novel major facilitator superfamily (MFS) transporter, named as TgApMFS1, is identified in the apicoplast. Depletion of TgApMFS1 significantly affects the growth of the parasites and impair the replication of the apicoplast genome. Further investigation on the role of TgApMFS1 for fatty acid synthesis and sugar metabolism indicate that deletion could lead to metabolic disorder in parasites.

## 2. Materials and Methods

Gene identification. The transmembrane domains in the sequences were analyzed by DeepTMHMM-1.0 (https://services.healthtech.dtu.dk/services/DeepTMHMM-1.0/, Version: 1.0, Manufacturer: Technical University of Denmark, Kongens Lyngby, Denmark, accessed on 26 June 2025). The conserved domains were identified by Conserved Domain Search (https://www.ncbi.nlm.nih.gov/Structure/cdd/wrpsb.cgi, Developer/Manufacturer: National Center for Biotechnology Information, Bethesda, MD, United States, accessed on 26 June 2025). The three-dimensional structure of TGGT1_309580 predicted by AlphaFold3 (https://alphafoldserver.com/, Developer/Manufacturer: DeepMind, London, UK, accessed on 26 June 2025) and presented in PyMOL3.0.3.

Parasites and cells. The split Cre recombinase (Cre-60 and Cre-59) fused with FRB and FKBP, respectively, was linked with a Crm cassette between them; a T2A peptide was cloned together with pBS-DHFR-TUB230, and the resulting plasmid was used to transfect Ku80- RH strain parasites. The parasites were then selected against chloramphenicol used as a parental strain (DiCre strain) to construct the rapamycin regulatable strain of the target gene.

The parasites were cultured on the hTERT-immortalized foreskin fibroblast cell line BJ-5ta (ATCC^®^ CRL-4001) in Dulbecco’s Modified Eagle Medium (DMEM) + 10% Fetal Bovine Serum (FBS).

Reagents and antibodies. Mouse and rabbit anti-TgSAG2 antibodies (1:1000) used for IFA (Immunofluorescence assay) and western blotting were prepared in our laboratory [14]. Tag antibodies were used at the following dilutions: Mouse monoclonal antibodies against HA (Sigma-Aldrich, St. Louis, MO, MI, USA, H9658): 1:1000 (IFA); Rabbit monoclonal antibodies against HA (Cell Signaling Technology, Danvers, MA, USA, 3724S): 1:1000 (IFA); Rabbit monoclonal antibodies against flag (Biodragon, Beijing, China, B1020): 1:1000 (IFA). Alexa Fluor 488 (Invitrogen, A11001/A11034) and Alexa Fluor 594 (Invitrogen, Carlsbad, CA, USA, A11037/A11032) were used as secondary antibodies (1:1000 for IFA). Anti-TgCPN60 rabbit antibody (1:500) were kept in our laboratory [15].

Plasmid construction. Two gRNA sequences specific for TGGT1_309580 (TgApMFS1-F-gRNA-F; TgApMFS1-F-gRNA-F, Table S1) were designed by the EuPaGDT web tool (http://grna.ctegd.uga.edu/, Developer/Manufacturer: Center for Tropical & Emerging Global Diseases University of Georgia, Athens, GA, USA, accessed on 26 June 2025) and cloned into the pCD-Cas9 [16] to construct the CRISPR/Cas9 plasmids. The identification primers are listed in Table S1.

Transfection and selection of parasites. The C terminal fragment of target gene flanked by 40-bp homologous arms and fused with a 3 × HA tag was amplified by PCRs. The amplification primers are listed in Table S1. This fragment was co-transfected with the CRISPR/Cas9 corresponding plasmid to construct the rapamycin-regulatable strain.

Immunofluorescence and microscopy. Cells grown in 35 mm dishes were fixed with 4% formaldehyde (Biosharp, Hefei, China, BL302A) in PBS for 15 min at room temperature and permeabilized with 0.3% Triton X-100 (Solarbio Life Sciences, Beijing, China, T8200) in phosphate buffered saline (PBS) for 30 min. Non-specific binding was blocked by incubating the samples with 5% bovine serum albumin (BSA; Solarbio Life Sciences, T8020) in PBS for 1 h at room temperature. Primary antibodies, diluted in blocking buffer, were applied and incubated overnight at 4 °C. After incubation, the cells were washed 5 times with Tris-buffered saline (TBS; 20 mM Tris-HCl, pH 8.0, 150 mM NaCl [Amresco, Solon, OH, USA, 0497; Sinopharm Chemical Reagent, Shanghai, China, 10019328]) containing 0.5% Tween-20 (Amresco, Solon, OH, USA, 0777). Subsequently, the samples were incubated with fluorophore-conjugated secondary antibodies and DAPI for nuclear staining. Confocal imaging was performed using either an LSM880 or LSM980 microscope (Zeiss, Oberkochen, Baden-Württemberg, Germany) equipped with a 100×/1.4 oil objective. Image acquisition and processing were conducted using Zen Blue 2.3 Lite software (Zeiss, Oberkochen, Baden-Württemberg, Germany) [17].

Intracellular replication assay. The cells were then fixed with 4% paraformaldehyde and probed with an anti-TgSAG2 antibody. The number of parasites per vacuole was determined, with 100 vacuoles examined per condition. Results are from three independent biological replicates.

Plaque assays. Monolayers of BJ-5ta grown in 12-well plates were inoculated with 500 tachyzoites per well and then cultured with or without rapamycin. After 36 h, the culture medium was replaced with fresh 5% FBS medium, and the cells were cultured continuously for another 7 days. The culture was then fixed with cold methanol and stained with Coomassie Brilliant Blue G250 (Solarbio Life Sciences, Beijing, China, C8430-5).

Heme abundance in the parasites. The parasites were cultured with rapamycin, while the control group was not supplemented with rapamycin for 48 h. After that, the culture dish was placed in an ice bath for 5 min. The number of parasites in the suspension was counted. To remove any residual culture medium, pre-cooled PBS was used to wash the parasites three times at 4 °C. The precipitate was then re-suspended in 400 μL of PBS and treated with ultrasound (Duty = 20%, Pulse on = 10 s, Pulse off = 30 s, Cycle = 6). Then, 100 μL of the sample was mixed with 900 μL of 2 mM oxalic acid solution in a brown EP tube and boiled or not boiled for 30 min. The emission intensity was measured using a multi-functional enzyme meter (Excitation: 400 nm, Emission: 608 nm). The fluorescence intensity for each group was calculated by subtracting the unboiled group’s intensity from that of the boiled group.

GC-MS analysis of fatty acids and sugar metabolites in the parasites. Cell culture dishes were washed with PBS and glucose-free DMEM medium containing 8 mM of 13C-labeled glucose was added to the cell culture dishes. The parasite strain of TgApMFS1^loxp^ was treated with rapamycin for 48 h. The cells were broken with a 27-G needle and filtered through a 5 µm filter. The suspension was then centrifuged at 4 °C at 1000× *g* for 10 min and washed three times with cold PBS. The parasite pellets (6 × 10^9^ parasites) were treated with liquid nitrogen for 10 min and subsequently stored at −80 °C.

GC-MS analysis was performed by Gereal Biotech in Shanghai, China. The fatty acids were extracted with a chloroform/methanol/water solution (in a 2:1:1 *v*/*v*/*v* ratio). After centrifugation, 600 µL of the lower phase was evaporated to dryness under a nitrogen stream and then redissolved in 50 µL of methanol. The extracts were chromatographically separated using an Ultimate 3000 UHPLC system (Thermo Fisher Scientific, Waltham, MA, USA) equipped with a BEH C18 column (100 mm × 2.1 mm, 1.7 µm, Waters) operating at a flow rate of 0.35 mL/min. The eluents were analyzed using a Q Exactive Hybrid Quadrupole-Orbitrap Mass Spectrometer (Thermo Fisher Scientific, Waltham, MA, USA) in heated electrospray ionization negative mode.

For the analysis of sugar metabolites, the samples were processed through five cycles of ultrasonication (1 min each) with 1 min intervals in an ice-water bath, followed by incubation at −20 °C for 30 min. Subsequently, samples were centrifuged at 15,000 rcf for 15 min at 4 °C. The supernatant (1 mL) was evaporated to dryness under a gentle nitrogen stream. The residues were reconstituted in 50 μL of 50% aqueous acetonitrile (1:1, *v*/*v*) prior to analysis. Chromatographic separation was performed on the ThermoFisher Ultimate 3000 UHPLC system equipped with the Waters BEH Amide column with a flow rate at 0.35 mL/min. Eluents were analyzed using the Q Exactive™ mass spectrometer (Thermo Fisher Scientific, Waltham, MA, USA) in heated electrospray ionization negative mode.

## 3. Results

### 3.1. Establishment of a Conditional Knockout Strain for TgApMFS1

The putative major facilitator superfamily transporter (TGGT1_309580) is identified in the apicoplast through the hyperplexed localization analysis of organelle proteins (hyperLOPIT) [18]. Recent studies have confirmed the localization of this transporter in the apicoplast [11,12], but its role in metabolism and parasite survival has yet to be explored. This transporter is named TgApMFS1 in this study to distinguish it from other reported members of the MFS family in the apicoplast.

**Figure 1:**
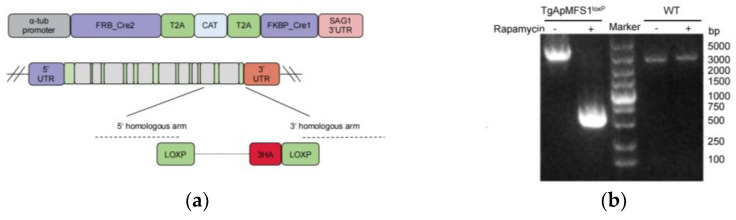

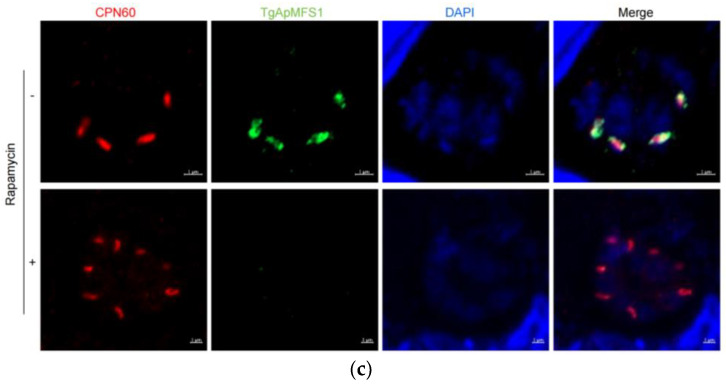
Construction of a conditional knockout strain of TgApMFS1. (**a**) A schematic diagram for the construction of a conditional knockout strain of TgApMFS1; (**b**) PCR identification of the excision of TgApMFS1 locus upon treatment with rapamycin; (**c**) The expression of TgApMFS1 examined using IFA. TgApMFS1 were stained with a rabbit anti-HA monoclonal antibody (green), and a polyclonal antibody against TgCPN60 was used to mark the apicoplast (red). Scale bar = 1 µm.

The fitness score of this gene is very low [19], suggesting that it may be essential. We decided to investigate the function of TgApMFS1 i parasite survival using a DiCre system. In this system, the genomic sequence flanked by loxP sites can be excised by the Cre recombinase upon treatment with rapamycin. To investigate the function of TgApMFS1 in parasite survival, two loxp fragments were inserted into the genomic locus of relative genes in the parasites expressing a split Cre recombinase (Figure 1a). The proper insertion of the loxp sites was confirmed through sequencing analysis (Figure 1b). The subcellular localizations of TgApMFS1 in the apicoplast was detected with anti-HA tag antibodies using immunofluorescence assays. The results indicated that TgApMFS1 clearly localized with the TgCPN60 at the apicoplast (Figure 1c). After 48 h treatment, the fluorescence signal of the targeting transporters could no longer be observed in the IFA analysis (Figure 1c).

### 3.2. TgApMFS1 Is Required for the Survival of T. gondii

**Figure 2:**
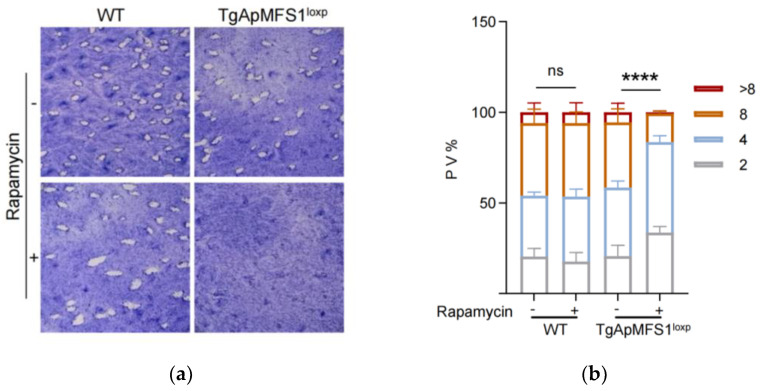
TgApMFS1 is required for the survival of parasites. (**a**) Plaque assays were performed by infecting BJ-5ta cells with TgApMFS1^loxp^ parasites for 8 days in the presence or absence of rapamycin. WT, wild type; (**b**) Replication of TgApMFS1^loxp^ parasites. The parasites were treated with or without rapamycin for 72 h, then inoculated into BJ-5ta cells in 12-well plates and allowed to grow with or without rapamycin for 24 h. The average number of parasites per vacuole was determined. The data were analyzed by a two-way ANOVA; **** *p* < 0.0001.

The role of TgApMFS1 in the growth and fitness of parasites was examined by treating the parasites with rapamycin using plaque assays. As expected, the wild-type parasites can form plaques both with and without rapamycin. However, the TgApMFS1^loxp^ parasites only form plaques in the absence of rapamycin, and not when treated with rapamycin. The results indicated that the depletion of TgApMFS1 severely impaired the lytic cycle of the parasites (Figure 2a). Next, we investigated the intracellular replication ability of the TgApMFS1-deficient parasites. The parasites were treated with rapamycin for 72 h in cell culture. After treatment, the egressed parasites were inoculated into a fresh cell monolayer and continued to be cultured for another 24 h in the presence of rapamycin. We found that the depletion of TgApMFS1 hindered the replication of the parasites at this time point (a total of 96 h of rapamycin treatment) (Figure 2b). These results indicated that TgApMFS1 is essential for the survival of parasites.

### 3.3. TgApMFS1 Is Required for the Inheritance of the Apicoplast

**Figure 3:**
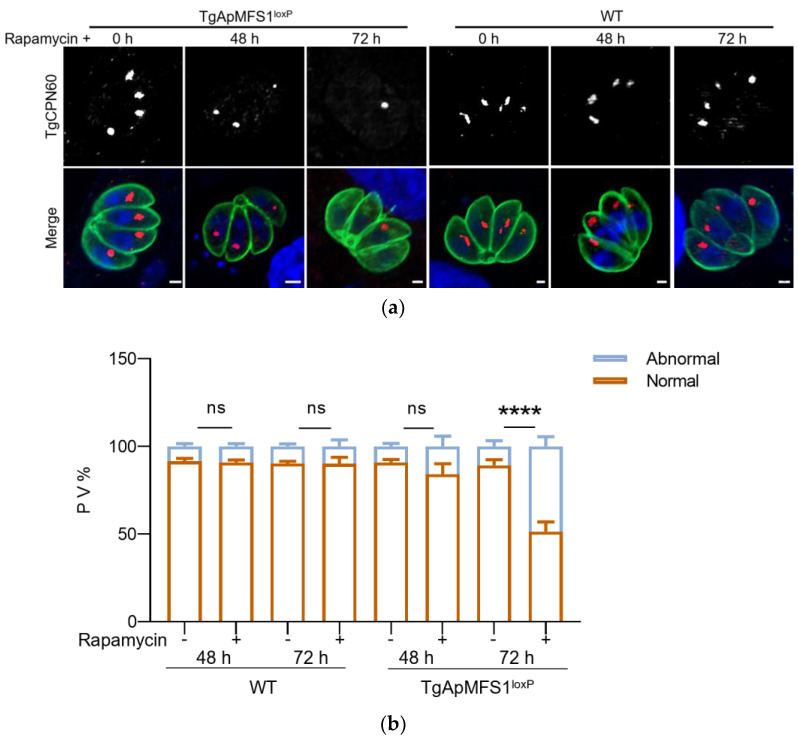
TgApMFS1 is required for the apicoplast inheritance. (**a**) The inheritance of the apicoplast was monitored in the parasites without TgApMFS1 by IFA. The parasites were treated as indicated in the figure. The parental DiCre strain served as the control (WT). A polyclonal antibody against TgCPN60 was used to label the apicoplast (red), while the parasites were stained with a mouse anti-TgSAG2 antibody (green). Scale bar = 1 µm; (**b**) The PVs in which the apicoplast was not observed in one or more parasites (abnormal) or where all apicoplasts could be observed (normal) were counted in (**a**). **** *p* < 0.0001.

To investigate whether TgApMFS1 is essential for the inheritance of the apicoplast, we pre-treated the recombinant parasites with rapamycin for 24 or 48 h. The egressed parasites were then inoculated into fresh cell culture and continued to be cultured for an additional 24 h in the presence of rapamycin. Subsequently, they were subjected to IFA analysis to assess the inheritance of the apicoplast in the daughter cells. We observed that the failure of apicoplast inheritance in the daughter parasites was evident only at 72 h (48 + 24), not at 48 h (24 + 24), after rapamycin treatment (Figure 3).

### 3.4. Depletion of TgApMFS1 Disturb the Fatty Acid Synthesis of the Parasites

**Figure 4:**
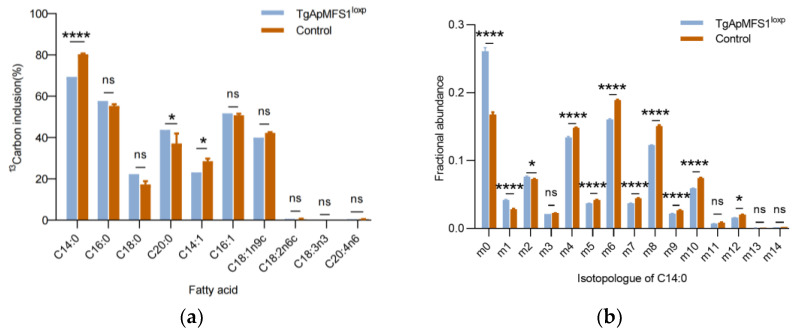
Depletion of TgApMFS1 disturbs fatty acid synthesis in the parasites. (**a**) GC-MS analysis of fatty acids in parasites lacking TgApMFS1. BJ-5ta cells were cultured with 13C-glucose for 6 h, inoculated with parasites, and then cultured with rapamycin for an additional 48 h. The egressed parasites were collected, purified using a 5 µm filter, and analyzed by GC-MS. The wild type parasites cultured with rapamycin were used as control in this study; **** *p* < 0.0001, * *p* = 0.0136, * *p* = 0.035; (**b**) Mass isotopologue distributions (MID) of fatty acid (FA) (C14:0) from ^13^C-glucose in the parasites lacking TgApMFS1. The *x*-axis indicates the number of ^13^C atoms in each Fatty Acid Methyl Ester (FAME), where ‘m0′ indicates the monoisotopic mass containing no ^13^C atoms, while ‘mX’ represents that mass with ‘X’ 13C atoms incorporated. **** *p* < 0.0001, **** *p* < 0.0001, * *p* = 0.0143, **** *p* < 0.0001, **** *p* < 0.0001, **** *p* < 0.0001, **** *p* < 0.0001, **** *p* < 0.0001, **** *p* < 0.0001, **** *p* < 0.0001, * *p* = 0.013.

The roles of TgApMFS1 in the FASII pathway in parasites were then assessed by gas chromatography-mass spectrometry (GC-MS) analysis. To facilitate this analysis, we added 13C-glucose to the cell culture allowing for its uptake by the host cells before the parasites were inoculated. The results showed that a wide range of fatty acids were labeled in the parasites (Figure 4a). After 48 h of treatment with rapamycin, the abundance of C14:0 fatty acids in TgApMFS1-deficient parasites reduced significantly compared with the control group (Figure 4a). The labelling of the C14:0 is uniform as observed by further analysis of mass isotopologue distributions (MID) (Figure 4b).

### 3.5. Depletion of TgApMFS1 Hinder the Sugar Metabolism

**Figure 5:**
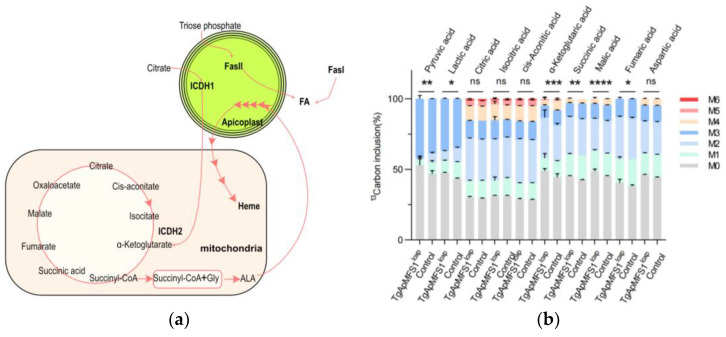
Depletion of TgApMFS1 affects the TCA cycle in the parasites. (**a**) A schematic diagram of the metabolic connection between the apicoplast and the mitochondria. (**b**) GC-MS analysis of sugar metabolites in the parasites lacking TgApMFS1. The parasites were cultured with rapamycin for 48 h. The wild type parasites cultured with rapamycin were used as control in this study. ‘m0′ indicates the monoisotopic mass containing no ^13^C atoms, while ‘mX’ represents that mass with ‘X’ 13C atoms incorporated. ** *p* = 0.0011, * *p* = 0.0214, *** *p* = 0.0002, ** *p* = 0.001, **** *p* < 0.0001, * *p* = 0.0513.

It is believed that α-ketoglutarate synthesized in the apicoplast plays a role in the TCA cycle (Figure 5a). However, the involvement of the apicoplast in sugar metabolism has not yet been evaluated. To investigate this, we assessed the metabolites in the sugar metabolic pathway after depleting TgApMFS1 in the parasites. The Bj-5ta cells were labeled with 13C-glucose for 6 h before the parasites were inoculated into the cell culture. The parasites were cultured for an additional 48 h in the presence of rapamycin. Subsequently, the egressed parasites were collected, filtered through 5 µm filters, and analyzed for metabolites using GC-MS analysis. Our findings revealed that the TCA cycle was significantly disrupted when TgApMFS1 was knocked out, resulting in a notable reduction of α-ketoglutarate, succinic acid, malic acid, fumaric acid, and others in the parasites treated with rapamycin for 48 h (Figure 5b).

### 3.6. Depletion of TgApMFS1 Disturbs the Heme Synthesis and Obstructs the Replication of the Apicoplast Genome in the Parasites

**Figure 6:**
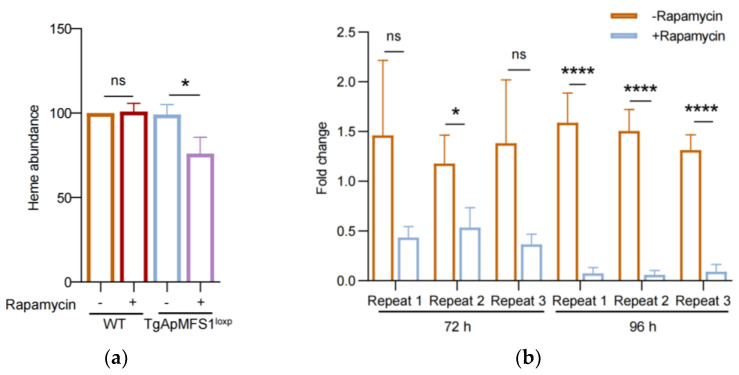
Depletion of TgApMFS1 affects the heme synthesis and the replication of apicoplast genome. (**a**) Heme abundance in the parasites lacking TgApMFS1. * *p* = 0.023; (**b**) The replication of the apicoplast genome in TgApMFS1^loxp^ parasites after rapamycin treatment were determined by real-time PCRs. * *p* = 0.033, **** *p* < 0.0001, **** *p* < 0.0001, **** *p* < 0.0001.

Furthermore, the apicoplast is known to play a role in heme synthesis (Figure 5a). We examined the levels of heme in parasites deficient in TgApMFS1. The results showed that the depletion of TgApMFS1 leads to a significant reduction in the total heme levels in the parasites (Figure 6a). The results indicated that the depletion of TgApMFS1 disrupted the metabolism of sugars and heme.

The apicoplast harbors a highly reduced genome that encodes genes primarily required for its replication and expression. The materials needed for the replication of this genome are transported from the cytoplasm of the parasites. However, the specific transporter responsible for this task is not yet known. In this study, we examined the replication of the apicoplast genome in parasites treated with rapamycin using real-time PCR. To do this, parasites were treated with rapamycin for 48 h. After egressing, the parasites were inoculated into a fresh BJ-5ta cell monolayer for an additional 24 or 48 h of culture, followed by real-time PCR analysis. We found that the depletion of TgApMFS1 resulted in a significant reduction of organellar genomic DNA at 72 h (48 + 24) treatment. After the parasites were treated for a total of 96 h (48 + 48) with rapamycin, we observed an even more severe impairment in the replication of the apicoplast genome (Figure 6b). These results indicated that TgApMFS1 contributes to the replication of the apicoplast genome in *T. gondii*.

## 4. Discussion

Over the past few decades, significant progress has been made in understanding the metabolic pathways involving the apicoplast. However, the molecular mechanisms that govern the transport of metabolic substances in the apicoplast remain very limited. Given the essentiality of the apicoplast for the survival of the parasite, it is important to identify novel essential transporters. In this study, we identified a new apicoplast transporter and evaluated its roles in parasite survival. The data in the current study could enhance our understanding of the mechanisms involved in molecule transportation and organelle biogenesis.

Translocation of nuclear-encoded apicoplast proteins is normally mediated by a N-terminal bipartite targeting sequence [20]. The signal peptides of these proteins are essential for entering into the endoplasmic reticulum (ER), while the transit peptide is responsible for targeting proteins to the apicoplast. However, some proteins lack both a signal peptide and a transit peptide [21,22]. This suggests the existence of an unknown pathway for transporting apicoplast proteins [15,23]. Bioinformatic analysis has shown that TgApMFS1 does not contain a signal peptide, indicating that it may be transported through this unidentified pathway. It would be worthwhile to investigate the sorting signals within TgApMFS1 that facilitate its translocation and whether the secretory pathway is involved in moving this protein to the apicoplast.

Our results indicated that the depletion of TgApMFS1 caused a wild range of deficiency in apicoplast-related metabolic pathways. This includes impaired synthesis of fatty acids and heme, as well as disrupted sugar metabolism. Therefore, these phenotypes are most likely the secondary effects caused by the loss of the apicoplast. Overall, these findings demonstrate that this transporter is essential for the biogenesis of the organelle.

According to our data, the deficiency of TgApMFS1 leads to a severe replication failure of the apicoplast genome. Therefore, it is reasonable to speculate that TgApMFS1 contributes to the genome replication, possibly by transporting the metabolites essential for the housekeeping functions. It is believed that transporters for metabolites, such as amino acids, nucleotides, cofactors, and vitamins essential for housekeeping functions, exist within the apicoplast. Additionally, important substances like thiamine and biotin, which are necessary for fatty acid synthesis, as well as oxidized forms of NAD^+^ and NADP^+^, citric acid, and CoA, also need to be transported into the apicoplast to meet metabolic requirements [3]. However, the molecular mechanisms involved in these transport processes remain unknown. The MFS family is the largest group of secondary active transporters. MFS transporters can transport a wide variety of substrates, including sugars, amino acids, organic and inorganic ions, vitamins, nucleobases, nucleosides, and nucleotides [24]. However, it is still necessary to identify the specific substrates of this transporter.

## Data Availability

The original contributions presented in this study are included in the article. Further inquiries can be directed to the corresponding author.

## Supplementary Materials

The following supporting information can be downloaded at: https://www.mdpi.com/article/10.3390/pathogens14080763/s1, Table S1: Primers used in the article.

## Abbreviations

The following abbreviations are used in this manuscript:

TgApMFS1	*T. gondii* apicoplast MFS transporter 1
GC-MS	gas chromatography-mass spectrometry
IFA	immunofluorescence assay
TgCPN60	*T. gondii* Chaperonin 60
DMEM	dulbecco’s modified eagle medium
FBS	fetal bovine serum
PfiTPT	*P. falciparum* innermost triosephosphate/phosphate transporter
PfoTPT	*P. falciparum* outermost triosephosphate/phosphate transporter
TgAPT1	*T. gondii* apicoplast phosphate transporter 1
ER	endoplasmic reticulum
IPP	isopentenyl pyrophosphate
MEP	methylerythritol phosphate
LPA	lysophosphatidic acid
FA	fatty acids
gRNA	guide RNA
PCR	polymerase chain reaction
TBS	Tris-buffered saline
TgSAG2	*T. gondii* surface antigen 2
hyperLOPIT	hyperplexed localization analysis of organelle proteins
MID	mass isotopologue distributions
FAME	fatty ccid methyl ester
TCA	tricarboxylic acid cycle
